# Impact of level of neonatal care on phlebotomy and blood transfusion in extremely low birthweight infants: a prospective, multicenter, observational study

**DOI:** 10.3389/fped.2023.1238402

**Published:** 2023-09-01

**Authors:** Ratchada Kitsommart, Anchalee Limrungsikul, Numtip Tongsawang, Nirucha Thamwiriyakul, Areeya Deesomchok, Nuttanan Pithakton, Bosco Paes

**Affiliations:** ^1^ Division of Neonatology, Department of Pediatrics, Faculty of Medicine Siriraj Hospital, Mahidol University, Bangkok, Thailand; ^2^ Division of Neonatology, Department of Pediatrics, Faculty of Medicine Ramathibodi Hospital, Mahidol University, Bangkok, Thailand; ^3^ Department of Pediatric Nursing, Faculty of Medicine Ramathibodi Hospital, Mahidol University, Bangkok, Thailand; ^4^ Department of Pediatrics, Chonburi Hospital, Chonburi, Thailand; ^5^ Department of Pediatrics, Buddhachinaraj Hospital, Phitsanulok, Thailand; ^6^ Department of Pediatrics (Neonatal Division), McMaster University, Hamilton, ON, Canada

**Keywords:** bronchopulmonary dysplasia, extremely-preterm infants, level of neonatal care, phlebotomy loss, transfusion

## Abstract

**Objectives:**

To explore the level of neonatal care on cumulative phlebotomy loss (cPL) and red cell transfusions in extremely low birthweight [ELBW; birthweight (BW) <1,000 g] infants, up to 40 weeks post-conceptual age (PCA). The secondary objective was to determine the associations between cPL and number of transfusions and between transfusions and hospital outcomes.

**Methods:**

A prospective, comparative, observational study was conducted in two level IV and two level III neonatal intensive care units (NICUs) in Thailand. Daily cPL volume and number of blood tests were recorded. Descriptive data are reported as frequency and percentage for categorical variables and median [25th percentile (P25), 75th percentile (P75)] for continuous data according to the data distribution. A *p*-value <0.05 was considered statistically significant.

**Results:**

210 ELBW infants were included; 99 and 111 were admitted to level IV and level III NICUs, respectively. Birth weight of level IV infants was lower 780.0 [660.0, 875.0] vs. 865.0 [723.0, 930.0] g; *p* < 0.001]. Initial group hematocrits were similar (43.1% vs. 44.0%, *p* = 0.47). cPL for each infant was 28.1 [16.5, 46.4] ml. Level IV infants had more tests (*n* = 89 [54, 195] vs. 59 [37, 88], *p* < 0.001). Counterintuitively, there was a lower cPL trend in level IV infants, but this was insignificant (19.6 [12.3, 52.3] vs. 28.9 [19.3, 45.3] ml; *p* = 0.06). The number of transfusions in both NICUs was similar 4 [2, 6], with a strong correlation between cPL and number of transfusions (*r* = 0.79, *p *< 0.001). Transfusions were significantly associated with bronchopulmonary dysplasia [BPD; adjusted RR (95% CI): 2.6 (1.2, 5.3), *p* = 0.01].

**Conclusions:**

Level IV NICUs conducted more blood tests in ELBW infants without a difference in cPL, and number of transfusions. Cumulative PL correlated with number of transfusions and was associated with BPD risk. Minimizing cPL by point-of-care tests and restrictive transfusion criteria, may reduce need for transfusion.

## Introduction

Extremely low birthweight (ELBW) infants (birthweight <1,000 g) are at-risk for anemia from incomplete erythropoiesis, short red cell life span, and low erythropoietin levels (1, 2). In addition, maternal complications such as antepartum hemorrhage may lead to a lower red cell mass. Severe anemia results in decreased tissue oxygen delivery that is associated with adverse outcomes such as impaired brain development (3) and necrotizing enterocolitis (NEC) (4, 5). Although the association of anemia with certain complications in preterm infants such as intraventricular hemorrhage (IVH) (6), apnea (7), increased cardiac workload (8), and poor growth (9) has been described, current evidence does not support the association of low hemoglobin concentrations in ELBW infants and long-term outcomes (7, 10, 11).

In the presence of significant anemia, physicians tend to administer red cell transfusions to improve oxygen delivery and physiological stability (12–14). Studies report that the rate of at least one red cell transfusion in ELBW infants is greater than 90% (11, 15). Red cell transfusions expose infants to pro-inflammatory cytokines and free iron which may result in transfusion-related adverse events such as retinopathy of prematurity (ROP), bronchopulmonary dysplasia (BPD), NEC, impaired neurodevelopmental outcomes, and mortality (15–23). Therefore, several postnatal strategies have been adopted to improve red cell volume through delayed umbilical cord clamping, early use of recombinant erythropoietin, tolerance of low hemoglobin levels, and prevention of further blood loss (15, 24). Although restrictive hemoglobin thresholds lead to a lower number of transfusions and donor exposure, the overall impact on clinical outcomes such as brain injury, NEC, BPD, or ROP, have been inconsequential (25). Attention has now been devoted to minimize the severity of anemia in this vulnerable population by decreasing the number of blood tests that reduce phlebotomy loss (PL) and the use of non-invasive monitoring (26).

ELBW infants are prone to significant PL due to their size and illness that requires intensive monitoring and medical care. Respiratory and hemodynamic instability in ELBW infants results in repetitive blood testing to facilitate supportive care during early postnatal life, particularly in infants who are critically ill. Unsurprisingly, this becomes a major source of blood loss over time.

There is no universal guidance for red cell transfusion in neonates. Transfusion protocols are mostly institution-based and are not uniform. They are mostly influenced by postnatal age, degree of illness, level of respiratory support (15, 27–29), and individual patients’ physiologic status (8, 30, 31). It is uncertain whether the level of intensive care and having written protocols affect the amount of PL or transfusion practice. Level IV neonatal intensive care units (NICUs) that provide broad subspecialist care, in a highly academic environment, may generate more blood work for meticulous monitoring than level III NICUs.

Our primary objective was to explore whether the level of neonatal care impacts cumulative phlebotomy loss (cPL) and number of red cell transfusions in ELBW infants up to 40 weeks post-conceptual age (PCA). The secondary objective was to determine the associations between cPL and number of transfusions, and between transfusions and hospital outcomes.

## Material and methods

This was a multicenter, comparative, prospective cohort study. ELBW infants (birthweight <1,000 g) who were admitted between February 1st, 2020 to April 30th, 2021, were enrolled in four NICUs in Thailand. The institutions included: Siriraj Hospital (Si) and Ramathibodi (RM) Hospital located in Bangkok in the center of Thailand which provide level IV neonatal care, and two other level III centers at Chonburi (CB) Hospital in the East of Thailand and Buddhachinnaraj (BC) Hospital in the North. The definitions of the levels of tertiary and quaternary care align with both the former and more recent American Academy of Pediatrics guidelines (32, 33). All NICU admissions are overseen solely by neonatologists. Relevant clinical parameters, volume of each phlebotomy loss, types of blood work, and red cell transfusions were collected daily from admission until 40 completed weeks postconceptional age or discharge from hospital, whichever came first. The study protocol was approved by the local institutional ethics committee of each study center. Patient recruitment was commenced following each institutional IRB approval (Si and RM from February 1st, 2020 and April 1st, 2020, respectively, CB from November1st, 2020, and BC from June 1st, 2020) until April 30th, 2021. The study size was a convenient, prospective sample over the study period. All data were collected anonymously in the case record form using only coded study numbers to identify individual participants during data collection.

Inclusion criteria comprised: (1) birthweight less than 1,000 g; (2) admission to the study centers within 72 h of life; and (3) parental written consent. Infants requiring unusual blood investigations or had fetal anemia requiring antenatal therapy, hydrops fetalis, surgical conditions that involved early surgery, lethal congenital anomalies, and critical congenital heart diseases were excluded. None of the study sites use erythropoietin for the prevention of anemia or for neuroprotection. Clinical practices in all 4 centers are similar and include umbilical vessel catheterization and insertion of peripheral arterial and central lines. Decisions to perform blood work, type of investigations, and red cell transfusions were at the discretion of attending physicians’ and local policy in each center. Transcutaneous blood gas and end-tidal carbon dioxide monitoring are selectively employed only at Si. There is no national guidance regarding types and frequency of blood work in ELBW infants in Thailand. Generally, a complete blood count, blood glucose, blood gas analysis and blood culture is sampled upon NICU admission. Blood chemistry is customarily performed on the second day of life. Thyroid function tests are done on days 3, 14 and 28 following birth and a screen for metabolic bone disease (serum alkaline phosphatase, phosphate, and calcium levels), is performed at 4 weeks of life. Additional blood work is dependent on an infant's respiratory and hemodynamic status and physicians’ discretion. Both level IV NICUs have institutional protocols for blood transfusion, unlike the level III units, and analyze blood gas and biochemistry specimens with point-of-care devices (i-STAT 1 Analyzer, Abbott Point of Care Inc., Illinois, USA Both level III NICUs (CB and BC) utilize their respective hospital laboratory services for all blood analyses. Supplementary Table S1 shows the hemoglobin (g/dl) threshold for red cell transfusion relative to the level of hospital care and respiratory support.

### Definitions

Hospital outcomes were evaluated in each study center by each local coinvestigator. Diagnosis of bronchopulmonary dysplasia (BPD) during the study period was based on the NICHD 2018 definition; radiographic confirmation of persistent parenchymal lung disease plus either room air or fractional inspired oxygen at various flow rates through nasal cannula to maintain arterial oxygen saturation in the 90%–95% range for ≥3 consecutive days, at 36 weeks post-conceptual age (34). NEC was diagnosed clinically by the attending staff with radiological confirmation. Standardized screening protocols for ROP and IVH by cranial ultrasonography were followed in the study centers.

Volume of PL was recorded uniformly in all the units during the study period. Blood volume drawn for arterial and venous samples were recorded and volume requirements for specific tests were dependent on local NICU policy. We estimated the blood volume for capillary samples as 0.005 ml/drop (35) and 0.02 ml/drop via direct venous puncture using a straight needle with the hub removed, and a drop-by-drop collection technique (36). Capillary tube volumes for blood gas analysis were estimated at 0.14 ml according to the manufacturer's catalogue (37). Blood was collected from arterial lines using two syringes. The dilute content of the first syringe was returned after a blood sample was obtained for analysis. Blood collections for a bundle of investigations were recorded as a single collection. Volume of blood transfusion was calculated using the infant's weight on the day of transfusion and typically 15 ml/kg was administered.

### Statistical analysis

Descriptive data are presented as frequency and percentage for categorical variables and median [25th percentile (P25), 75th percentile (P75)] for continuous data according to the data distribution. Missing values for each variable were excluded in the analysis. Variables were compared using chi-square or Fisher-exact test for categorical variables, and Mann-Whitney *U* test for continuous variables with non-normal distribution. Correlation between cPL and number of transfusions was calculated using Pearson correlation coefficient. The association between red cell transfusion and major morbidities was explored using risk ratio, with 95% confidence interval (CI) adjusted for study centers and other potential confounders. All statistical analyses were performed using SPSS Statistics version 18.0 (SPSS, Inc., Chicago, IL, USA) and Stata software version 14. A *p*-value <0.05 was considered statistically significant.

## Results

During the study period, 210 ELBW infants were enrolled. Ninety-nine and 111infants were admitted to the level IV and level III NICUs, respectively. Table 1 outlines maternal and infants’ demographic characteristics. Generally, there were no significant differences in maternal characteristics. Infants admitted to the level IV NICUs were significantly smaller (median [P25, P75] birthweight was 780.0 [660.0, 875.0] vs. 865.0 [723.0, 930.0] g; *p* < 0.001] and comprised a higher proportion of twins [34 (34.3%) vs. 13 (11.7%); *p* < 0.001] than those in the level III NICUs. Despite having significantly lower 5-minute Apgar scores (7 [5, 8] vs. 8 [6, 9], *p* = 0.01), infants in the level IV NICUs were less likely to receive mechanical ventilation compared to the level III NICUs [56 (56.6%) vs. 96 (86.5%); *p* < 0.001]. The initial hematocrit was similar between the groups (43.1% vs. 44.0%, *p *= 0.47). None of the infants underwent major surgical procedures or had cardiac surgery performed.

**Table 1:** Maternal and infant demographic characteristics.

	Total (*n* = 210)	Level IV NICUs (*n* = 99)	Level III NICUs (*n* = 111)	*p* *
Maternal characteristics (*n* = 197)				
Age (year)	30.5 [27.0, 34.0]	32.0 [27.3, 35.8]	29 [24.8, 34.0]	0.01*
Primigravida	90 (45.9)	42 (47.7)	48 (44.4)	0.65
Gravida	2 (1, 2)	2 (1, 2.8)	2 (1, 2)	0.67
Parity	0 (0, 1)	0 (0, 1)	0 (0, 1)	0.17
Diabetes	20 (10.2)	6 (6.8)	14 (12.8)	0.16
Hypertension	55 (27.9)	20 (22.7)	35 (32.1)	0.14
Antepartum hemorrhage	11 (5.6)	2 (2.3)	9 (8.3)	0.12
Maternal infection	20 (10.2)	7 (8.0)	13 (11.9)	0.48
Cesarean section	128 (65.0)	62 (70.5)	66 (60.6)	0.15
Infants’ characteristics (*n* = 210)				
Gestational age (weeks)	27 [25, 28]	26 [25, 28]	27.0 [26, 29]	0.05
Male sex	101 (48.1)	50 (50.5)	51 (45.9)	0.51
Birth weight (g)	820.0 [703.8, 910.0]	780.0 [660.0, 875.0]	865.0 [723.0, 930.0]	<0.001*
Twins	47 (22.4)	34 (34.3)	13 (11.7)	<0.001*
5-minute Apgar score	8 [6, 9]	7 [5, 8]	8 [6, 9]	0.01*
Small-for-gestational age	46 (22.0)	27 (27.3)	19 (17.1)	0.08
Mechanical ventilation	152 (72.4)	56 (56.6)	96 (86.5)	<0.001*
Initial hematocrit (%)	43.2 [38.5, 49.9]	43.1 [39.1, 48.6]	44.0 [37.0, 52.3]	0.47
Delayed cord clamping	16 (7.6)	16 (16.2)	0	<0.001*
Days of birth hospitalization	78.5 [60.0, 102.8]	87.0 [65.0, 123.0]	71.0 [57.0, 96.0]	0.005*
Hospital death	28 (13.3)	13 (13.1)	15 (13.5)	0.94

Data are presented as number (percentage) or median [25th, 75th percentile].

* *p* value indicates difference in the variables between infants admitted into level IV NICUs and level III NICUs. A *p* value <0.05 is statistically significant.

**Figure 1:**
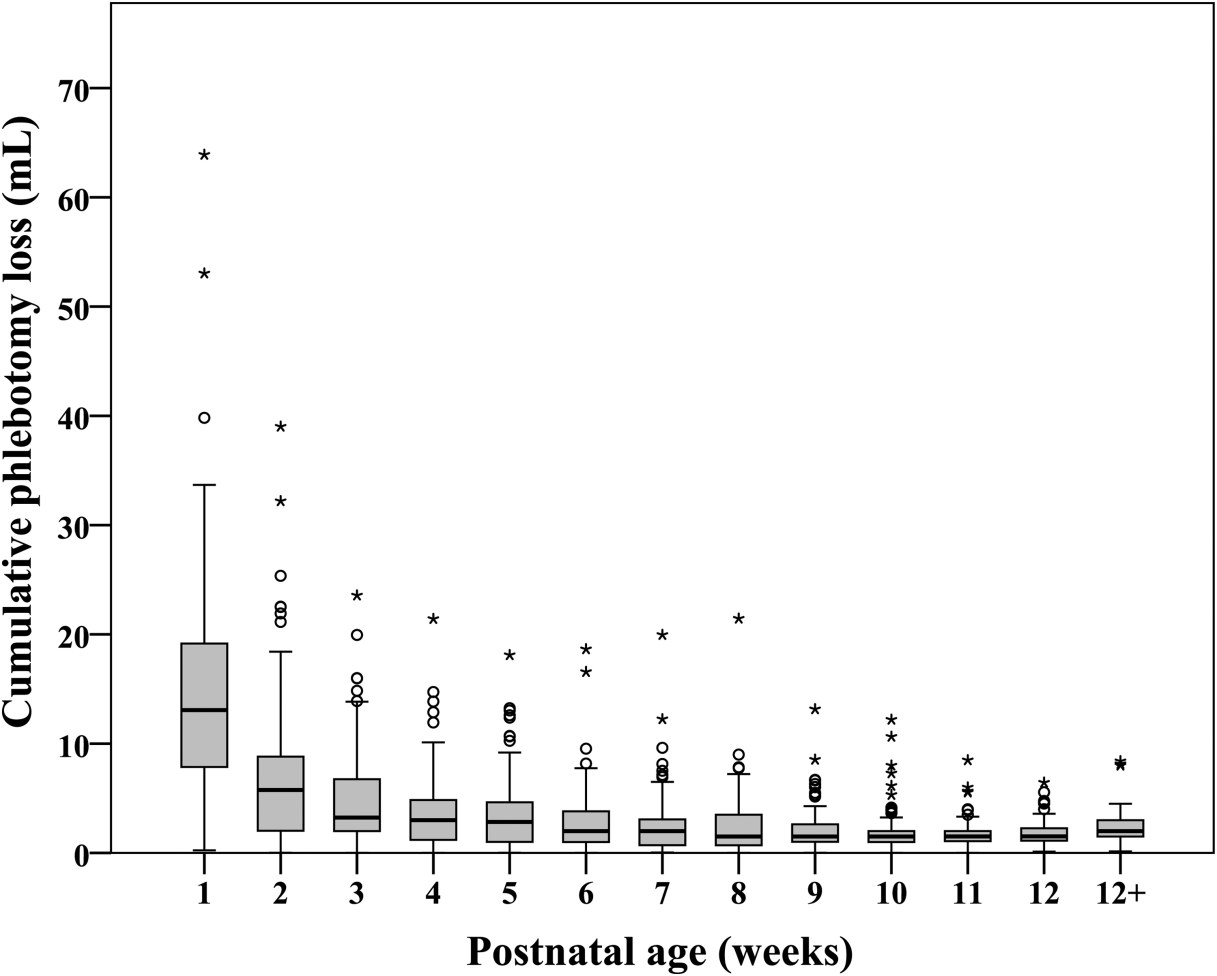
Distribution of cumulative phlebotomy loss in extremely low birthweight infants from birth until 40 weeks postconceptional age (*N* = 210).

**Figure 2:**
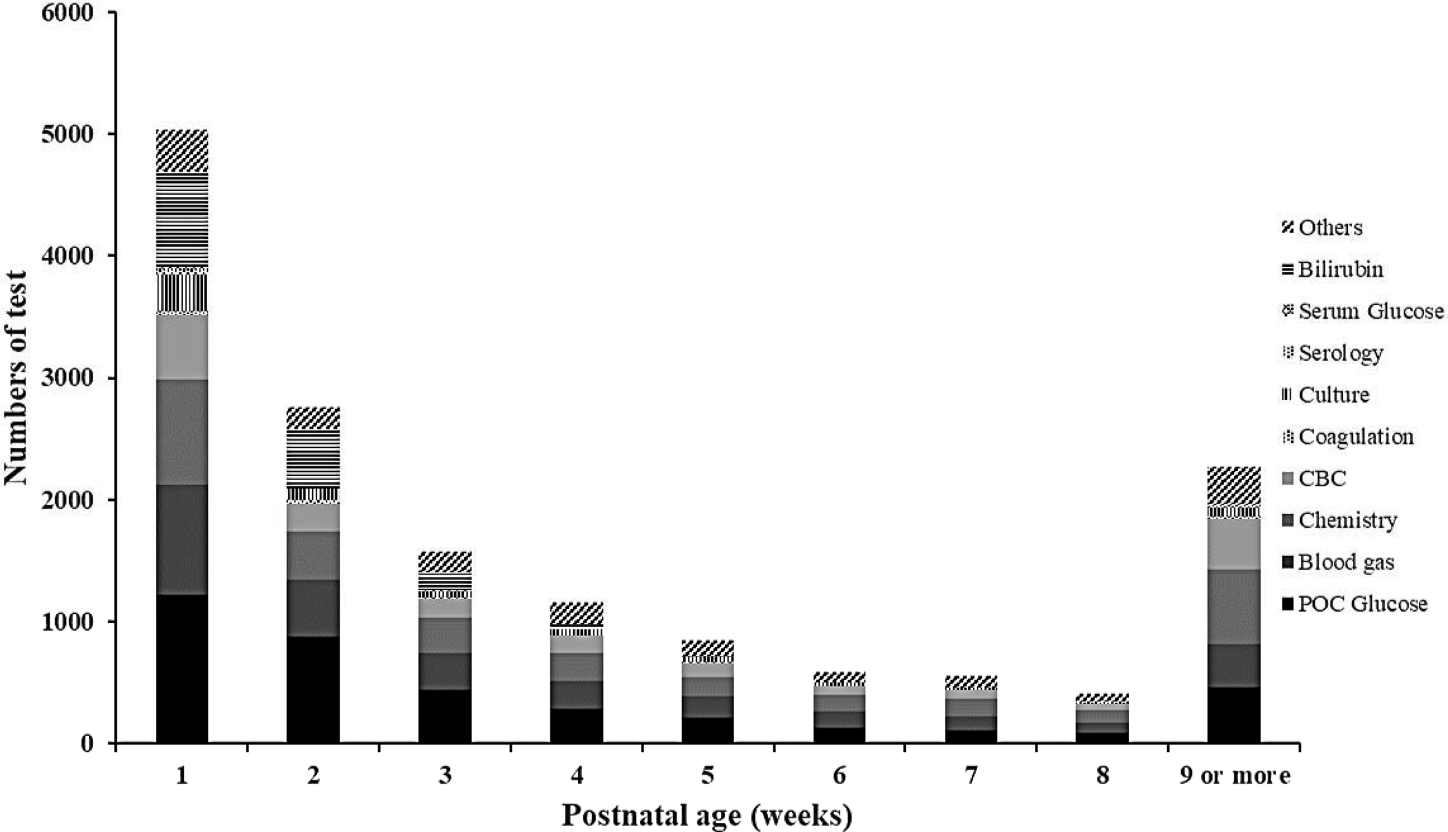
Frequency and types of blood tests in extremely low birthweight infants from birth until 40 weeks postconceptional age (*N* = 210). CBC, complete blood count; POC glucose, point-of-care glucose.

Figure 1 shows the distribution of cPL per infant over time. Median PL [P25, P75] within 40 weeks PCA for each infant was 28.1 [16.5, 46.4] ml. Cumulative PL occurred mainly during the first 4 weeks of life and was 13.1 [7.9, 19.2], 5.8 [2.0, 8.8], 3.2 [2.0, 6.8], and 3.0 [1.2, 4.9] ml for each infant from the first until the fourth week of life, respectively. Table 2 shows the cPL from admission until 40 weeks PCA and Figure 2 indicates the types of blood work conducted for each postnatal week. Level IV NICUs had more blood tests than the level III NICUs (89 [54, 195] vs. 59 [37, 88], *p *< 0.001). Blood glucose, blood gas, and chemistry were the three most common types of blood tests performed. Generally, infants in the level IV NICUs had a higher number of tests than the level III NICUs, particularly for blood glucose, blood gas, chemistry, and coagulation, that were statistically significant. The type of blood test that incurred the highest cPL was blood chemistry (9.0 [6.0, 16.0] ml), complete blood count (CBC; 4.5 [2.5, 7.5] ml), and blood gas analysis (3.5 [1.0, 6.6] ml), respectively. Hence, there was a trend toward lower cPL in the level IV NICUs compared to the level III NICUs, but this was statistically insignificant (19.6 [12.3, 52.3] vs. 28.9 [19.3, 45.3] ml; *p* = 0.06).

**Table 2:** Cumulative phlebotomy loss per infant and transfusions from birth until 40 weeks postconceptional age.

	Total (*N* = 210)	Level IV NICUs (*n* = 99)	Level III NICUs (*n* = 111)	*p* *
Cumulative phlebotomy loss per infant (ml)	28.1 [16.5, 46.4]	19.6 [12.3, 52.3]	28.9 [19.3, 45.3]	0.06
Number of tests per infant	67 [45, 110]	89 [54, 195]	59 [37, 88]	<0.001*
Blood glucose (*n* = 209)	22 [12, 38.5]	33 [20, 61]	14 [9, 23.3]	<0.001*
Blood gas (*n* = 198)	13 [4.8, 24]	16 [4, 70]	11 [5, 20]	0.03*
Chemistry (*n* = 207)	12 [8, 19]	15.5 [9, 27.3]	9 [7, 14]	<0.001*
Complete blood count (*n* = 209)	6 [4, 10]	7 [3.8, 12.3]	6 [4, 8]	0.22
Blood culture (*n* = 205)	2 [1, 4]	2 [1, 4]	3 [2, 4]	0.07
Coagulation screen (*n* = 42)	1 [1, 3]	2 [1, 4]	1 [1, 1.5]	0.04*
Serology (*n* = 44)	1 [1, 1]	1 [1, 1]	1 [1, 2]	0.07
Others (*n* = 208)	13 [9, 18]	13 [8, 18]	14 [10, 18]	0.22
Number of transfusions (*n* = 181)	4 [2, 6]	3 [1, 7]	4 [2, 5]	0.72
Total transfusion volume (ml/kg) (*n* = 181)	50.0 [30.0, 90.0]	52.0 [27.0, 112.3]	45.0 [30.0, 82.6]	0.17
Postnatal day of first transfusion (*n* = 181)	5 [2, 14]	4 [2, 11]	6 [3, 17]	0.15

Data are presented as number (percentage) or median [25th, 75th percentile].

* *p* value indicates difference in variables between infants admitted to level IV NICUs and level III NICUs. A *p* value <0.05 is statistically significant.

**Figure 3:**
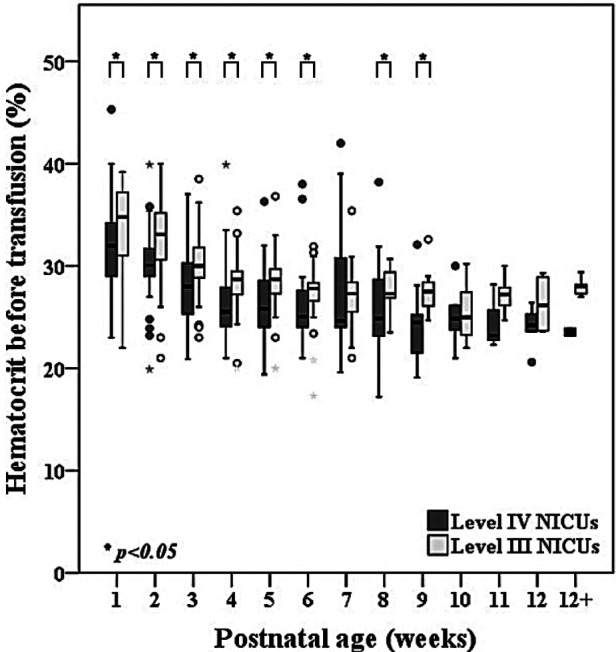
Comparisons of hematocrit level between level IV and level III NICUs before red cell transfusion in extremely low birthweight infants from birth until 40 weeks postconceptional age (*n* = 181).

One-hundred and eighty-one infants (86.2%) received at least 1 transfusion and of these, 110 infants (60.8%) received a transfusion during the first week of life. The median [P25, P75] number of transfusions was 4 [2, 6] times for each infant which was similar between the level IV and level III NICUs. Of the 364 transfusions in the level IV NICUs, 285 (78.3%) were guided by the hematocrit level. For the remaining 79 transfusions, 42 (53.2%) were administered during mechanical ventilation and 20 (25.3%) during non-invasive ventilation. Total transfusion volume and postnatal age of the first transfusion was similar between the groups. Notably, the median volume of transfusion within the first 2 weeks was 30 [15, 55] ml/kg which was 50% of the total transfused volume within the 40 weeks PCA. Figure 3 shows the hematocrit level before each transfusion over time across the level IV and level III NICUs. The hematocrit before transfusion in the level IV NICUs was significantly lower compared to the level III NICUs.

**Figure 4:**
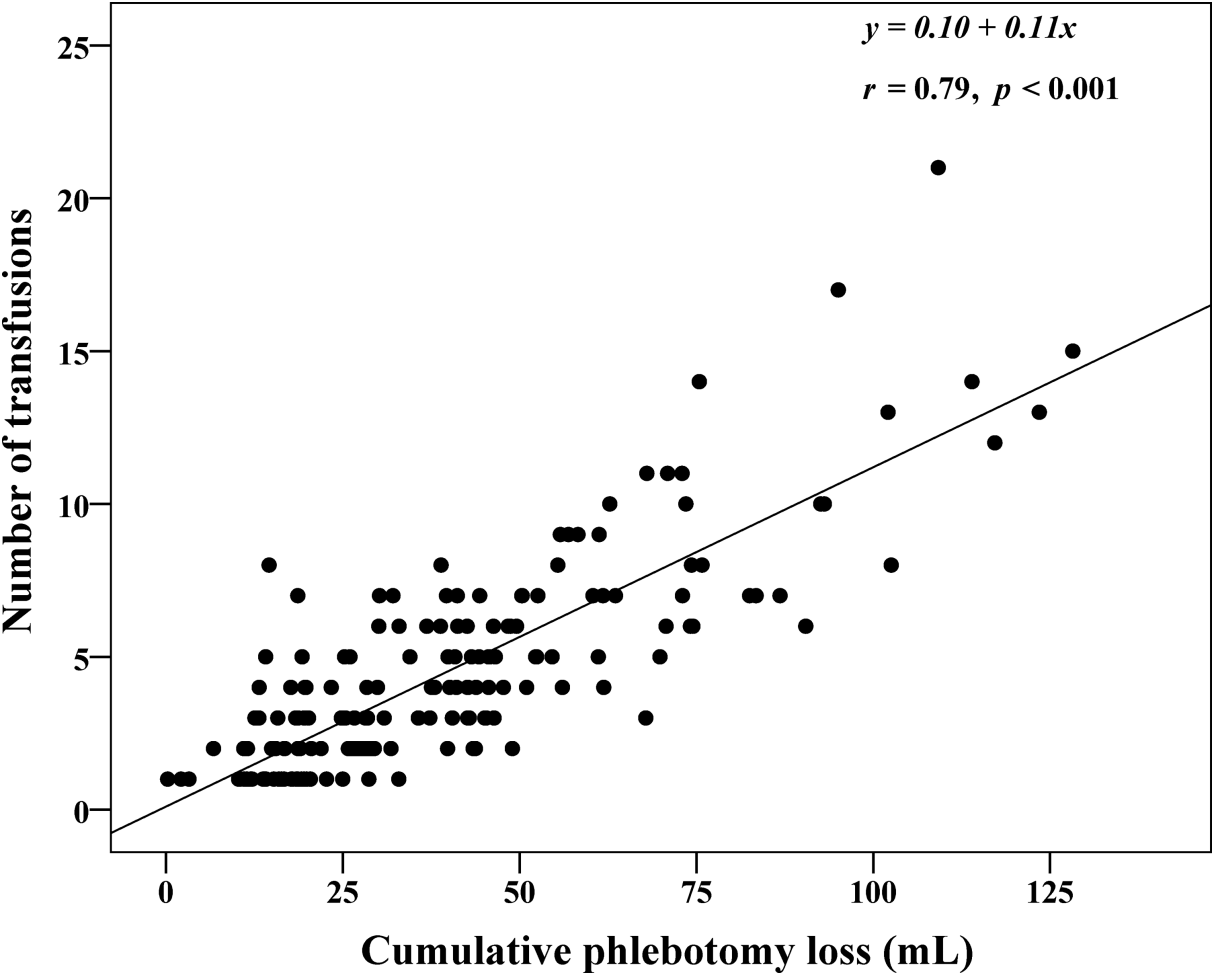
Correlation between cumulative phlebotomy loss and number of red cell transfusions in extremely low birthweight infants from birth until 40 weeks postconceptional age (*N* = 210).

Figure 4 shows a strong positive correlation between the cPL and number of transfusions (*r* = 0.79, *p *< 0.001). Table 3 outlines factors associated with the number of transfusions within the 40-week time period. After adjustment for confounding covariates and study sites, lower gestational age, birthweight, initial hematocrit, and less cPL were significantly associated with lower number of transfusions. Supplementary Table S2 presents maternal and infant demographic characteristics of ELBW infants who received and did not receive blood transfusion. Table 4 shows the association between blood transfusion and hospital outcomes. After adjusting for study centers and mechanical ventilation, blood transfusion was significantly associated with bronchopulmonary dysplasia [adjusted RR (95% CI): 2.6 (1.2, 5.3), *p* = 0.01]. The rates of NEC [adjusted RR (95% CI): 1.3 (0.4, 4.4), *p* = 0.68], IVH [adjusted RR (95% CI): 1.6 (0.8, 2.9), *p* = 0.16], ROP [adjusted RR (95% CI): 1.7 (0.8, 3.6), *p* = 0.15], and death [adjusted RR (95% CI): 0.8 (0.3, 2.0), *p* = 0.57] were statistically insignificant.

**Table 3:** Factors associated with number of transfusions in extremely preterm infants until 40 weeks postconceptional age (*n* = 181).

	Univariate linear regression		Multivariate linear regression	
	Beta	*p*	Beta	*p*
Written transfusion protocol	0.2 (−0.8, 1.3)	0.65		
Gestational age (weeks)	−0.6 (−0.8, −0.4)	<0.001*	−0.2 (−0.3, −0.01)	0.04*
Birthweight (g)	−0.006 (−0.009, −0.002)	0.002*	−0.003 (−0.005, −0.002)	0.03*
Twins	0.2 (−1.0, 1.5)	0.72		
5-minute Apgar score	−0.4 (−0.6, −0.1)	0.001*	0.1 (−0.04, 0.2)	0.17
Mechanical ventilation	1.9 (0.7, 3.0)	0.002*	0.2 (−0.6, 1.0)	0.64
Initial hematocrit (every percentage increment)	−0.05 (−0.09, −0.01)	0.02*	−0.04 (−0.06, −0.01)	0.004*
Delayed cord clamping	−3.3 (−5.6, −1.0)	0.005*	−0.4 (−1.8, 1.1)	0.607
Cumulative phlebotomy loss (every 1 ml increment)	0.11 (0.10, 0.12)	<0.001*	0.12 (0.10, 0.13)	<0.001*
Maternal infection	−0.6 (−2.2, 1.0)	0.46		
Cesarean section	−0.6 (−1.7, 0.4)	0.24		

Multivariate linear regression was adjusted for confounding covariates in demographic characteristics and the study sites.

* *p* < 0.05 is statistically significant.

**Table 4:** Association between received blood transfusion and hospital morbidity.

	No transfusion (*n* = 29)	Received transfusion (*n* = 181)	Adjusted relative risk **(**95**%** CI**)**	*p*
Necrotizing enterocolitis	3/28 (10.7)	28/172 (16.3)	1.3 (0.4, 4.4)	0.68
Bronchopulmonary dysplasia	6/27 (22.2)	110/168 (65.5)	2.6 (1.2, 5.3)	0.01*
Intraventricular hemorrhage	8/21 (38.1)	59/107 (55.1)	1.6 (0.8, 2.9)	0.16
Retinopathy of prematurity	6/20 (30.0)	63/108 (58.3)	1.7 (0.8, 3.6)	0.15
Death	4/29 (13.8)	24/181 (13.3)	0.8 (0.3, 2.0)	0.57

Relative risk was adjusted for study centers and mechanical ventilation.

* *p *< 0.05 is considered statistically significant.

## Discussion

Red cell transfusions improve oxygen delivery in sick neonates. However, there are substantial risks that result in short- and long-term morbidities and mortality (12, 15, 22). ELBW infants by virtue of their prematurity have physiological anemia. This state can be accentuated in sick preterm infants by chronic blood loss due to frequent phlebotomy, to ensure proper supportive care (38). The median cPL within the 40 weeks PCA of our cohort was 28.1[16.5, 46.4] ml, in which 13.1 [7.9, 19.2] ml (43%) and 5.8 [2.0, 8.8] ml (15.2%) occurred during the first and the second week of life. This estimated cPL aligns with the study by Counsilman et al. (24.2 [15.8, 30.3] ml/kg) (39) but is relatively low when compared to a median of 40 ml/kg during the first 2 weeks of life in the study by Hellstrom et al. (38) and 83 [70, 97] ml during the first 10 weeks of life in the study by Puia-Dumitrescu et al. (40) Although the time period and unit of estimation (ml or ml/kg) were different between the studies, in our cohort with a median [P25, P75] birthweight of 820.0 [703.8, 910.0] g, the cPL was lower. Notably, our median birthweight and cPL was comparable to the study by Counsilman et al. (835 [689, 1,086] g) (39), while the median birthweight was higher in the studies conducted by Hellstrom et al. [mean (SD) 797 (21.5) g] and Puia-Dumitrescu et al. (median 665 [587, 822] g), which reported higher cPL. Therefore, the variation in cPL could be partly related to ELBW and the need for more blood tests during the neonatal hospital stay. Nevertheless, it is also arguable that the estimation of cPL, particularly from capillary sampling, affects the volumes reported. Hellstrom et al. estimated PL from volumes required for each analysis (38). while Counsilman et al. estimated PL from routine requirements for each analysis (0.0625 ml/drop) (39). We prospectively estimated each PL from the actual amount of the sample collected and the volume of each drop from capillary or venous samples to get the closest estimation.

The lower cPL was even more pronounced in our level IV NICUs (median [P25, P75]; 19.6 [12.3, 52.3] ml) compared to other studies. Blood glucose, blood gas analysis and blood chemistry were the three most common tests performed, and the latter accounted for the highest cPL per infant. One-half of blood glucose tests were assayed from capillary samples which minimized overdraw volume compared to arterial and venous samples, which supports the benefit of point-of-care glucose testing in clinical practice (41). Although there is limited evidence for the use of non-invasive monitoring to minimize the needs for transfusion (42), Counsilman et al. (39) reported relatively small cPL in ELBW infants with the use of a bundled protocol based on in-line point-of-care monitoring, and strict blood volume collection. We support the strategy of non-invasive, transcutaneous blood gas and end-tidal carbon-dioxide monitoring to minimize routine blood tests in order to decrease cPL and need for transfusion.

As expected, level IV NICU infants had a significantly higher number of common tests (blood glucose, blood gas, and chemistry) than those in the level III NICUs (Table 2). This may be influenced by the academic environment which encourages close monitoring and more intensive investigation, while the level III NICUs tend to perform blood tests using physician discretion and infants’ clinical status. Nevertheless, cPL in the level IV NICUs group was lower, but statistically insignificant, than the level III NICUs (median and IQR was 19.6 [12.3, 52.3] vs.28.9 [19.3, 45.3] ml, respectively; *p* = 0.06). We attribute this finding to the use of point-of-care tests for blood chemistry and blood gas analysis in both level IV NICUs, which led to minimum cPL (43). Of note, arterial and venous sample volumes tend to be overdrawn compared to point-of-care tests, to avoid unexpected errors during analysis (44). Since majority of the cPL (58.3%) and transfusions (50%) occurred during the first 2 weeks of life, we scrutinized this period more closely. The number of tests from arterial sampling during the first 2 weeks of life was similar (23 [11, 55.5] vs. 20 [14, 26.3] for level IV and level III NICUs, respectively; *p *= 0.18) but level IV NICUs had significantly more venous (17 [12, 25] vs. 23 [17, 29]; *p* < 0.001) and capillary samples drawn (14 [10, 20.5] vs. 7 [5, 11]; *p* < 0.001) than the level III NICUs. This aligns with our findings of higher number of tests but similar volume between the two groups for the respective route of blood sampling.

Eighty-six percent of infants in our cohort received at least 1 red cell transfusion which was less than over 90% reported in other studies (11, 15, 39). Although there was no written protocol in the level III NICUs, their preferred practice for blood testing is restrictive. The institutional guidelines in both level IV NICUs are relatively similar with restrictive strategies for testing based on postnatal age and level of respiratory support. Overall adherence with the transfusion protocol in the level IV NICUs group was 78.3% which is relatively low compared to the 3.5% protocol violation rate in the transfusion of prematures (TOP) trial (11). Reasons for protocol deviations in our study were due to concerns of high cPL during the first week of life and intention to increase oxygen delivery during acute sickness such as sepsis or impending cardiac failure, frequent apnea, and pre-operatively. The average number of transfusions was 4 [2, 6] which also less than the mean (standard deviation) of 5.4 (4.8) in the preterm erythropoietin neuroprotection (PENUT) and the TOP trials (11, 15). Our median hematocrit levels before transfusion closely aligned with the protocol of the ETTNO trial (10). Hematocrit prior to transfusion subsequently decreased with advancing postnatal age from the median of 32.9% during the first week of life to 31.7%, 29.7%, 28% in the subsequent 2–4 weeks of life. Although infants in the level IV NICUs were generally transfused at a lower hematocrit than the level III NICUs (Figure 3), the number of transfusions and transfusion volume was similar between the groups (Table 2). Therefore, a restrictive transfusion threshold alone did not decrease the number or volume of transfusion in ELBW infants (45). Previous studies have inconsistently shown an association between cPL and need for transfusion (38, 41). We found a strong correlation between cPL and number of transfusions (*r *= 0.79, *p* < 0.001) (Figure 3). Therefore, we encourage the additional strategies of non-invasive monitoring, avoidance of unnecessary routine blood tests, and application of the microtechnique point-of-care testing to minimize cPL and reduce transfusions in ELBW.

Transfusion-related immunomodulation results from exposure to free-radical and pro-inflammatory cytokines following red cell transfusion in particularly vulnerable infants (16) and may cause NEC (46), BPD (38, 47), severe IVH (48, 49), ROP (17, 18), or death (23, 50). We found transfusions in our cohort was only associated with BPD which concurs with the study by Hellstrom et al. (38) and Bolat et al. (51). Although we adjusted for potential confounding factors, the association between transfusion and BPD remained significant. In a recent metanalysis of 21 studies, Tang et al. postulated several reasons in support of the association between BPD and red cell transfusions (52). These include increased hyperoxic exposure through adult hemoglobin, inflammatory mediators in transfused blood that may enhance free radical formation, infection, and fibrosis, iron overload that stimulates oxidative stress secondary to immature iron metabolism and an inadequate antioxidant defense mechanism especially in ELBW infants and cytokine-induced lung inflammation that may result in transfusion related acute lung injury (19, 53–57).

Variations in neonatal care are common in clinical practice (58). Since there is no uniform guideline on what blood tests should be performed in ELBW infants, between-center variation is inevitable and depends on institutional guidance or resides on the personal discretion of individual healthcare providers based on each patient's clinical condition. Our study raises awareness of iatrogenic blood loss which led to need for transfusions and the association of transfusion with BPD. Based on our findings of the higher number of tests conducted in level IV NICUs, we propose striking a balance between maintaining quality of care in ELBW infants while minimizing risks associated with transfusions, using noninvasive monitoring, point-of-care testing, and avoidance of routine and unnecessary blood work.

Several strengths and limitations of our study merit consideration. Our prospective study explored the impact of the level of neonatal care on cPL and number of transfusions. Neonatologists in all of the participating centers provide standardized care for ELBW infants that align with international guidelines, thereby affording validity and both internal and external generalizability of our results. Previous studies comprised relatively small sample sizes and were retrospective (38–40) which led to challenges in PL volume estimation. Each collection in our study involved various amounts of blood dependent on the route of sampling, volume requirement for each test, and avoidance of overdrawn volumes to reduce re-collection. Moreover, multiple tests were bundled into a single collection to decrease unnecessary testing. The prospective design facilitated a correct estimate of blood volume compared to previous retrospective studies. However, we realize that our volumes may be underestimated because we did not include small amounts of blood that may have been directly lost from the venous needle puncture site or absorbed into gauze during the collection or minimal unintentional overdraws. Nevertheless, the magnitude of cPL should be cautiously interpreted by level III and level IV NICUs in which blood tests may be solely performed using point-of-care devices rather than combined laboratory services as outlined in our protocol.

## Conclusion

Level IV NICUs conducted more blood tests in ELBW infants than the level III NICUs but there was no difference in cPL, as well as the number and volume of administered transfusions. Cumulative PL correlated with the number of transfusions and was associated with the risk for BPD. Strategies to minimize cPL by microtechnique point-of-care testing, noninvasive monitoring methods and restrictive transfusion criteria could potentially reduce the need for transfusion in ELBW infants.

## Data Availability

The raw data supporting the conclusions of this article will be made available by the authors, without undue reservation.

## References

[1] Strauss RG. Anaemia of prematurity: pathophysiology and treatment. Blood Rev. (2010) 24(6):221–5. 10.1016/j.blre.2010.08.001 20817366 PMC2981681

[2] Kuruvilla DJ Widness JA Nalbant D Schmidt RL Mock DM An G Estimation of adult and neonatal rbc lifespans in anemic neonates using rbcs labeled at several discrete biotin densities. Pediatr Res. (2017) 81(6):905–10. 10.1038/pr.2017.14 28099421 PMC5470643

[3] Singh G Wallin DJ Abrahante Llorens JE Tran PV Feldman HA Georgieff MK Dose- and sex-dependent effects of phlebotomy-induced anemia on the neonatal mouse hippocampal transcriptome. Pediatr Res. (2022) 92(3):712–20. 10.1038/s41390-021-01832-9 34775474 PMC9098692

[4] Arthur CM Nalbant D Feldman HA Saeedi BJ Matthews J Robinson BS Anemia induces gut inflammation and injury in an animal model of preterm infants. Transfusion. (2019) 59(4):1233–45. 10.1111/trf.15254 30897226 PMC6525338

[5] Ozcan B Aydemir O Isik DU Bas AY Demirel N. Severe anemia is associated with intestinal injury in preterm neonates. Am J Perinatol. (2020) 37(6):603–6. 10.1055/s-0039-1683982 30947347

[6] Dekom S Vachhani A Patel K Barton L Ramanathan R Noori S. Initial hematocrit values after birth and peri/intraventricular hemorrhage in extremely low birth weight infants. J Perinatol. (2018) 38(11):1471–5. 10.1038/s41372-018-0224-6 30206347

[7] Kirpalani H Whyte RK Andersen C Asztalos EV Heddle N Blajchman MA The premature infants in need of transfusion (pint) study: a randomized, controlled trial of a restrictive (low) versus liberal (high) transfusion threshold for extremely low birth weight infants. J Pediatr. (2006) 149(3):301–7. 10.1016/j.jpeds.2006.05.011 16939737

[8] Alkalay AL Galvis S Ferry DA Simmons CF Krueger RC Jr. Hemodynamic changes in anemic premature infants: are we allowing the hematocrits to fall too low? Pediatrics. (2003) 112(4):838–45. 10.1542/peds.112.4.838 14523175

[9] Keller A Hermanussen M Vogtmann C Kiess W Keller E. Effect of erythrocyte transfusion on longitudinal bone growth of premature infants assessed by mini-knemometry. Eur J Pediatr. (1999) 158(10):871–2. 10.1007/s004310051230 10486102

[10] Franz AR Engel C Bassler D Rüdiger M Thome UH Maier RF Effects of liberal vs restrictive transfusion thresholds on survival and neurocognitive outcomes in extremely low-birth-weight infants: the ettno randomized clinical trial. J Am Med Assoc. (2020) 324(6):560–70. 10.1001/jama.2020.10690 PMC7420159 32780138

[11] Kirpalani H Bell EF Hintz SR Tan S Schmidt B Chaudhary AS Higher or lower hemoglobin transfusion thresholds for preterm infants. N Engl J Med. (2020) 383(27):2639–51. 10.1056/NEJMoa2020248 33382931 PMC8487591

[12] Kalteren WS Verhagen EA Mintzer JP Bos AF Kooi EMW. Anemia and red blood cell transfusions, cerebral oxygenation, brain injury and development, and neurodevelopmental outcome in preterm infants: a systematic review. Front Pediatr. (2021) 9:644462. 10.3389/fped.2021.644462 33718309 PMC7952449

[13] Ibonia KT Bada HS Westgate PM Gomez-Pomar E Bhandary P Patwardhan A Blood transfusions in preterm infants: changes on perfusion index and intermittent hypoxemia. Transfusion. (2018) 58(11):2538–44. 10.1111/trf.14808 30291755 PMC6221924

[14] Jain D D'Ugard C Bancalari E Claure N. Cerebral oxygenation in preterm infants receiving transfusion. Pediatr Res. (2019) 85(6):786–9. 10.1038/s41390-018-0266-7 30587847

[15] Vu PT Ohls RK Mayock DE German KR Comstock BA Heagerty PJ Transfusions and neurodevelopmental outcomes in extremely low gestation neonates enrolled in the penut trial: a randomized clinical trial. Pediatr Res. (2021) 90:109–16. 10.1038/s41390-020-01273-w 33432157 PMC7797706

[16] Crawford TM Andersen CC Stark MJ. Effect of repeat transfusion exposure on plasma cytokine and markers of endothelial activation in the extremely preterm neonate. Transfusion. (2020) 60(10):2217–24. 10.1111/trf.15952 32710580

[17] Dani C Reali MF Bertini G Martelli E Pezzati M Rubaltelli FF. The role of blood transfusions and iron intake on retinopathy of prematurity. Early Hum Dev. (2001) 62(1):57–63. 10.1016/s0378-3782(01)00115-3 11245995

[18] Lust C Vesoulis Z Jackups R Jr Liao S Rao R Mathur AM. Early red cell transfusion is associated with development of severe retinopathy of prematurity. J Perinatol. (2019) 39(3):393–400. 10.1038/s41372-018-0274-9 30459388 PMC6391181

[19] Collard KJ. Is there a causal relationship between the receipt of blood transfusions and the development of chronic lung disease of prematurity? Med Hypotheses. (2006) 66(2):355–64. 10.1016/j.mehy.2005.04.046 16236459

[20] Christensen RD Lambert DK Henry E Wiedmeier SE Snow GL Baer VL Is “transfusion-associated necrotizing enterocolitis” an authentic pathogenic entity? Transfusion. (2010) 50(5):1106–12. 10.1111/j.1537-2995.2009.02542.x 20051059

[21] Mohamed A Shah PS. Transfusion associated necrotizing enterocolitis: a meta-analysis of observational data. Pediatrics. (2012) 129(3):529–40. 10.1542/peds.2011-2872 22351894

[22] Shah P Cannon DC Lowe JR Phillips J Christensen RD Kamath-Rayne B Effect of blood transfusions on cognitive development in very low birth weight infants. J Perinatol. (2021) 41(6):1412–8. 10.1038/s41372-021-00997-9 33911186 PMC8238787

[23] dos Santos AM Guinsburg R de Almeida MF Procianoy RS Leone CR Marba ST Red blood cell transfusions are independently associated with intra-hospital mortality in very low birth weight preterm infants. J Pediatr. (2011) 159(3):371–6e1-3. 10.1016/j.jpeds.2011.02.040 21489555

[24] Ohlsson A Aher SM. Early erythropoietin for preventing red blood cell transfusion in preterm and/or low birth weight infants. Cochrane Database Syst Rev. (2006) 3:Cd004863. 10.1002/14651858.CD004863.pub2 16856062

[25] Ibrahim M Ho SK Yeo CL. Restrictive versus liberal red blood cell transfusion thresholds in very low birth weight infants: a systematic review and meta-analysis. J Paediatr Child Health. (2014) 50(2):122–30. 10.1111/jpc.12409 24118127

[26] Su PC Chung HW Yang ST Chen HL. Effect of small volume blood sampling on the outcomes of very low birth weight preterm infants. Children. (2022) 9(8):1190. 10.3390/children9081190 36010080 PMC9406683

[27] Guillén Ú Cummings JJ Bell EF Hosono S Frantz AR Maier RF International survey of transfusion practices for extremely premature infants. Semin Perinatol. (2012) 36(4):244–7. 10.1053/j.semperi.2012.04.004 22818544 PMC3579510

[28] Patel RM Hendrickson JE Nellis ME Birch R Goel R Karam O Variation in neonatal transfusion practice. J Pediatr. (2021) 235:92–99.e4. 10.1016/j.jpeds.2021.04.002 33836184 PMC8316298

[29] Saito-Benz M Sandle ME Jackson PB Berry MJ. Blood transfusion for anaemia of prematurity: current practice in Australia and New Zealand. J Paediatr Child Health. (2019) 55(4):433–40. 10.1111/jpc.14222 30246273

[30] Hardy JF. Current status of transfusion triggers for red blood cell concentrates. Transfus Apher Sci. (2004) 31(1):55–66. 10.1016/j.transci.2004.06.002 15294196

[31] Banerjee J Aladangady N. Biomarkers to decide red blood cell transfusion in newborn infants. Transfusion. (2014) 54(10):2574–82. 10.1111/trf.12670 24797124

[32] American Academy of Pediatrics Comittee on Fetus Newborn, Barfield WD Papile L-A Baley JE Benitz W Cummings J Levels of neonatal care. Pediatrics. (2012) 130(3):587–97. 10.1542/peds.2012-1999 22926177

[33] Stark AR Pursley DM Papile LA Eichenwald EC Hankins CT Buck RK Standards for levels of neonatal care: ii, iii, and iv. Pediatrics. (2023) 151(6):e2023061957. 10.1542/peds.2023-061957 37212022

[34] Higgins RD Jobe AH Koso-Thomas M Bancalari E Viscardi RM Hartert TV Bronchopulmonary dysplasia: executive summary of a workshop. J Pediatr. (2018) 197:300–8. 10.1016/j.jpeds.2018.01.043 29551318 PMC5970962

[35] Grady M Pineau M Pynes MK Katz LB Ginsberg B. A clinical evaluation of routine blood sampling practices in patients with diabetes: impact on fingerstick blood volume and pain. J Diabetes Sci Technol. (2014) 8(4):691–8. 10.1177/1932296814533172 24876439 PMC4764211

[36] Bond MM Richards-Kortum RR. Drop-to-drop variation in the cellular components of fingerprick blood: implications for point-of-care diagnostic development. Am J Clin Pathol. (2015) 144(6):885–94. 10.1309/ajcp1l7dkmpchpeh 26572995

[37] Sarstedt AG & Co. KG. Blood gas analytics: collection systems for arterial, venous, and capillary sampling [cited August 23, 2023]. Available at: https://www.sarstedt.com/fileadmin/user_upload/99_Broschueren/NEU/478/20_478_0100_200_blutgas_analytik_1020.pdf

[38] Hellstrom W Forssell L Morsing E Savman K Ley D. Neonatal clinical blood sampling led to major blood loss and was associated with bronchopulmonary dysplasia. Acta Paediatr. (2020) 109(4):679–87. 10.1111/apa.15003 31505053 PMC7155086

[39] Counsilman CE Heeger LE Tan R Bekker V Zwaginga JJ te Pas Ab Iatrogenic blood loss in extreme preterm infants due to frequent laboratory tests and procedures. J Matern Fetal Neonatal Med. (2021) 34(16):2660–5. 10.1080/14767058.2019.1670800 31588840

[40] Puia-Dumitrescu M Tanaka DT Spears TG Daniel CJ Kumar KR Athavale K Patterns of phlebotomy blood loss and transfusions in extremely low birth weight infants. J Perinatol. (2019) 39(12):1670–5. 10.1038/s41372-019-0515-6 31582812 PMC7331095

[41] Widness JA Madan A Grindeanu LA Zimmerman MB Wong DK Stevenson DK. Reduction in red blood cell transfusions among preterm infants: results of a randomized trial with an in-line blood gas and chemistry monitor. Pediatrics. (2005) 115(5):1299–306. 10.1542/peds.2004-1680 15867038 PMC2867083

[42] Persad E Sibrecht G Ringsten M Karlelid S Romantsik O Ulinder T Interventions to minimize blood loss in very preterm infants-a systematic review and meta-analysis. PLoS One. (2021) 16(2):e0246353. 10.1371/journal.pone.0246353 33556082 PMC7870155

[43] Madan A Kumar R Adams MM Benitz WE Geaghan SM Widness JA. Reduction in red blood cell transfusions using a bedside analyzer in extremely low birth weight infants. J Perinatol. (2005) 25(1):21–5. 10.1038/sj.jp.7211201 15496875

[44] Lin JC Strauss RG Kulhavy JC Johnson KJ Zimmerman MB Cress GA Phlebotomy overdraw in the neonatal intensive care nursery. Pediatrics. (2000) 106(2):E19. 10.1542/peds.106.2.e19 10920175

[45] Keir A Pal S Trivella M Lieberman L Callum J Shehata N Adverse effects of red blood cell transfusions in neonates: a systematic review and meta-analysis. Transfusion. (2016) 56(11):2773–80. 10.1111/trf.13785 27600435

[46] Gephart SM. Transfusion-associated necrotizing enterocolitis: evidence and uncertainty. Adv Neonatal Care. (2012) 12(4):232–6. 10.1097/ANC.0b013e31825e20ee 22864004 PMC3414263

[47] Rashid N Al-Sufayan F Seshia MM Baier RJ. Post transfusion lung injury in the neonatal population. J Perinatol. (2013) 33(4):292–6. 10.1038/jp.2012.114 22955289

[48] dos Santos AM Guinsburg R de Almeida MF Procianoy RS Marba ST Ferri WA Factors associated with red blood cell transfusions in very-low-birth-weight preterm infants in Brazilian neonatal units. BMC Pediatr. (2015) 15:113. 10.1186/s12887-015-0432-6 26341125 PMC4560891

[49] Christensen RD Baer VL Lambert DK Ilstrup SJ Eggert LD Henry E. Association, among very-low-birthweight neonates, between red blood cell transfusions in the week after birth and severe intraventricular hemorrhage. Transfusion. (2014) 54(1):104–8. 10.1111/trf.12234 23672455

[50] Wang YC Chan OW Chiang MC Yang PH Chu SM Hsu JF Red blood cell transfusion and clinical outcomes in extremely low birth weight preterm infants. Pediatr Neonatol. (2017) 58(3):216–22. 10.1016/j.pedneo.2016.03.009 27514234

[51] Bolat F Dursun M Sariaydin M. Packed red blood cell transfusion as a predictor of moderate-severe bronchopulmonary dysplasia: a comparative cohort study of very preterm infants. Am J Perinatol. (2023). 10.1055/a-2051-8245 36898407

[52] Tang L Zhu TT Zhao J. Association between red blood cell transfusion and bronchopulmonary dysplasia: a systematic review and meta-analysis. Front Pediatr. (2023) 11:1095889. 10.3389/fped.2023.1095889 37325359 PMC10266411

[53] Ryan RM Ahmed Q Lakshminrusimha S. Inflammatory mediators in the immunobiology of bronchopulmonary dysplasia. Clin Rev Allergy Immunol. (2008) 34:174–90. 10.1007/s12016-007-8031-4 18330726

[54] Patel RM Knezevic A Yang J Shenvi N Hinkes M Roback JD Enteral iron supplementation, red blood cell transfusion, and risk of bronchopulmonary dysplasia in very-low-birth-weight infants. Transfusion. (2019) 59(5):1675–82. 10.1111/trf.15216 30801736 PMC6499698

[55] Zhang Z Huang X Lu H. Association between red blood cell transfusion and bronchopulmonary dysplasia in preterm infants. Sci Rep. (2014) 4(1):4340. 10.1038/srep04340 24614152 PMC3949297

[56] Keir AK McPhee AJ Andersen CC Stark MJ. Plasma cytokines and markers of endothelial activation increase after packed red blood cell transfusion in the preterm infant. Pediatr Res. (2013) 73(1):75–9. 10.1038/pr.2012.144 23095979

[57] Ming S Zhang D Chen L Shi Y. Effects of anemia and red blood cell transfusion in preterm infants on the development of bronchopulmonary dysplasia: a propensity score analysis. All Life. (2021) 14(1):830–9. 10.1080/26895293.2021.1972350

[58] Adams M Bassler D Bucher HU Roth-Kleiner M Berger TM Braun J Variability of very low birth weight infant outcome and practice in Swiss and US neonatal units. Pediatrics. (2018) 141(5):e20173436. 10.1542/peds.2017-3436 29654158

