# Supplementary material for: Impact of level of neonatal care on phlebotomy and blood transfusion in extremely low birthweight infants: a prospective, multicenter, observational study
Source: Front Pediatr. 2023 Sep 1;11:1238402. doi: 10.3389/fped.2023.1238402 (PMC10505442; doi:10.3389/fped.2023.1238402)
Supplement: Supplementary file 1 [file Table1.pdf]

Supplemental Table 1 Threshold hemoglobin (g/dL) for red cell transfusion relative to the level of hospital care and respiratory support

Postnatal week	No respiratory support				With respiratory support			
	Level IV		Level III		Level IV		Level IV	
	Si	RM	CB	BC	Si	RM	CB	BC
1	10.0	10.0	10.0	10.0	11.0	11.7	11.7	11.5
2	8.5	8.3	8.3	8.5	10.0	10.0	10.0	10.0
≥3	7.0	7.7	7.7	7.5	8.5	7.7	7.7	8.5

Si; Siriraj Hospital, RM; Ramathibodi Hospital, CB; Chonburi Hospital, BC; Buddhachinnaraj Hospital

Supplemental Table 2 Maternal and infant demographic characteristics in relation to transfusion

	Total (n=210)	Received transfusion (n =181)	No transfusion (n=29)	p^a
**Maternal characteristics** (n= 197)				
Age (year)	30.5 [27.0, 34.0]	34 [27.3, 37.5]	30 [26.0, 34.0]	0.05
Primigravida	90 (45.9)	13 (46.4)	77 (45.8)	0.95
Gravida	2 [1, 2]	2 [1, 2]	2 [1, 2]	0.91
Parity	0 [0, 1]	0 [0, 1]	0 [0, 1]	0.85
Diabetes	20 (10.2)	2 (7.1)	18 (10.7)	0.75
Hypertension	55 (27.9)	8 (28.6)	47 (27.8)	0.93
Antepartum hemorrhage	11 (5.6)	1 (3.6)	10 (5.9)	1.00
Maternal infection	20 (10.2)	1 (3.6)	19 (11.2)	0.32
Cesarean section	128 (65.0)	24 (85.7)	104 (61.5)	0.02[a]
**Infants' characteristics** (n= 210)				
Gestational age (weeks)	27 [25, 28]	28 [27, 29.5]	26 [25, 28]	0.002
Male sex	109 (51.9)	16 (55.2)	93 (51.4)	0.70
Birth weight (g)	820 [703.75, 910]	850 [710.50, 945]	805 [700, 906]	0.21
Twins	47 (22.4)	8 (27.6)	39 (21.5)	0.47
5-minute Apgar score	8 [6, 9]	8 [6, 9]	8 [6, 9]	0.65
Small-for-gestational age	46 (21.9)	10 (34.5)	36 (19.9)	0.08
Mechanical ventilation	152 (72.4)	13 (44.8)	139 (76.8)	<0.001[a]
Initial hematocrit (%)	43.15 [38.53, 49.85]	50.3 [46, 54.35]	42.6 [36.55, 49.28]	<0.001[a]
Delayed cord clamping	16 (7.6)	7 (24.1)	9 (5.0)	0.002[a]
Days of birth hospitalization	78.50 [60, 102.8]	65 [50, 81]	83 [61, 107.5]	0.01[a]
Hospital death	28 (13.3)	4 (13.8)	24 (13.3)	1.00

Data are presented as number (percentage) or median [25th, 75th percentile],
[a] *p* value indicates difference in the variables between infants who received and did not receive transfusion. A *p* value <0.05 is statistically significant
